# Lymph node tuberculosis after allogeneic haematopoietic stem cell transplantation: an atypical presentation of an uncommon complication

**DOI:** 10.3332/ecancer.2015.535

**Published:** 2015-05-05

**Authors:** Guillermo Martín-Sánchez, Marta Drake-Pérez, José Luis Rodriguez, Lucrecia Yañez, Andrés Insunza, Carlos Richard

**Affiliations:** 1 Hematology Department, Hospital Universitario Marqués de Valdecilla, Avda. Valdecilla s/n, Santander 39008, Spain; 2 Radiology Department, Hospital Universitario Marqués de Valdecilla, Avda. Valdecilla s/n, Santander 39008, Spain; 3 Pathology Department, Hospital Universitario Marqués de Valdecilla, Avda. Valdecilla s/n, Santander 39008, Spain

**Keywords:** lymph node tuberculosis, haematopoietic stem cell transplantation

## Abstract

Mycobacterium tuberculosis infections are uncommon complications in the haematopoietic stem cell post-transplant period. Most cases are reactivations of latent infections affecting the lung. We present an atypical case of isolated lymph node tuberculosis after an allogeneic haematopoietic stem cell transplantation, which highlights the importance of having a high suspicion index, even in non-endemic countries.

## Background

Although haematopoietic stem cell transplant (HSCT) recipients have an increased risk of infection, as a result of severe cellular immunity suppression, Mycobacterium tuberculosis infections are rare. The incidence is proportional to the prevalence in the surrounding population and ranges from less than 1% in non-endemic countries to 16% in Pakistan. The lung is the most commonly involved organ, and the mortality rate can be as high as 50% [1]. The largest series published to date, from Spain, reported an incidence of 0.41% among 2,866 allogeneic patients with HSCT [2]. We present a case of tuberculosis (TB) reactivation after an allogeneic HSCT with isolated lymph node involvement, which mimics other post-transplant complications and makes the diagnosis difficult.

## Case report

A 55-year-old male, with multiple myeloma, was admitted with a fever up to 38.5°C and cervical lymph node enlargement 70 days after an allogeneic HSCT from an unrelated donor with human leukocyte antigen DQB1 mismatch (9/10 matched), for early progression after an autologous HSCT. After reduced intensity conditioning with fludarabine (40 mg/m^2^ 4 days), melphalan (70 mg/m^2^ 1 day), and thymoglobulin (2.5 mg/kg 3 days), he received a T-cell-replete peripheral blood allograft containing 5.7 × 10^6^ CD34+ cells/kg. Graft-versus-host disease prophylaxis consisted of tacrolimus (from day 1) and methotrexate (on days +1, +3, and +6). He received acyclovir as anti-infective prophylaxis and was isolated in a HEPA filtered room during the severe neutropenia period. The patient denied previous exposure or infection by M. tuberculosis. Bacillus Calmette–Guerin vaccination status was unknown and tuberculin skin test (TST) is not routinely performed to HSCT candidates at our hospital.

**Figure 1.**
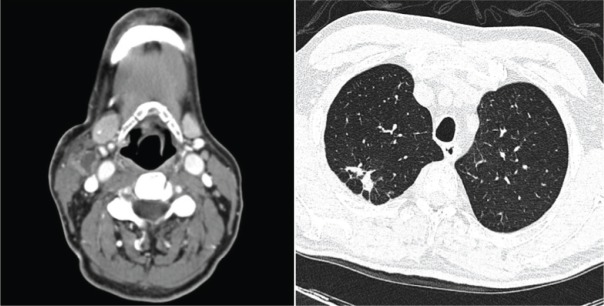
(A) Contrast enhanced CT on day +70 after HSCT presented right cervical lymph nodes enlargement with central necrosis. (B) Highresolution chest CT revealed old pulmonary scar lesions with pleural thickening in the right upper lobe.

At admission, a contrast-enhanced computed tomography (CT) showed right laterocervical lymph node enlargement (Figure 1A) and a high-resolution chest CT revealed old pulmonary scar lesions in the right upper lobe but no signs of active infection (Figure 1B). Quantitative polymerase chain reaction (qPCR) in the peripheral blood-detected epstein–barr viral (EBV) replication (820 copies/mL) and a first cervical biopsy was not diagnostic. Tacrolimus taper was started, and the patient was discharged afebrile with oral antibiotherapy.

**Figure 2.**
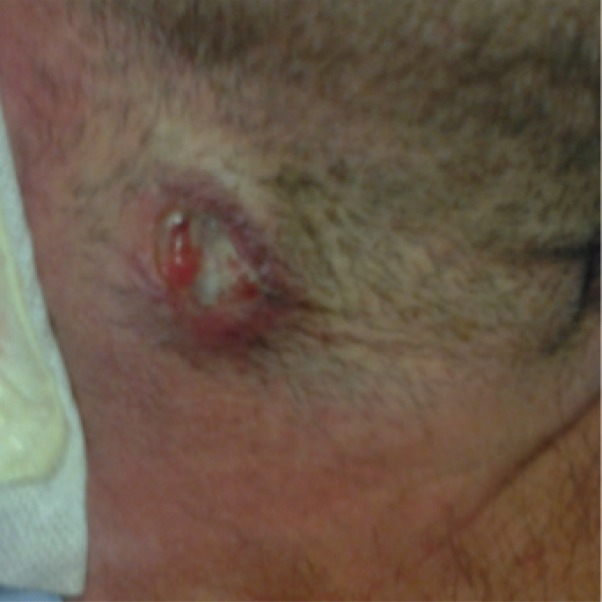
Cervical lymph node biopsy wound on day +90 with purulent suppuration.

**Figure 3.**
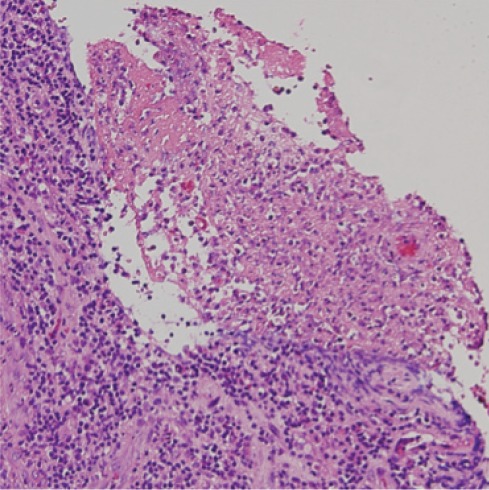
Haematoxylin and eosin stain of the lymph node showed granuloma formations with necrosis.

On day +90, fever was noted again, and the biopsy wound presented purulent suppuration (Figure 2), which was sent for culture. EBV replication was no more detected during the follow-up. Histologic exam of a second biopsy showed caseating granulomas (Figure 3). PCR analysis of the lymph node demonstrated the presence of M. tuberculosis consensus sequences, which was confirmed by microbiological cultures isolating M. tuberculosis complex. Combined treatment with rifampicin, isoniazid, and pyrazinamide was initiated with a quick recovery of all infectious symptoms. Antituberculosis therapy was maintained for 9 months with a complete resolution of tuberculosis (TB).

## Discussion

Tuberculosis (TB) can be developed by transmission from actively infected individuals but most of the cases in patients with cancer are due to reactivation, so effort should be directed to detecting latent infections and identifying those patients at risk [3]. Although our patient denied previous contact or infection, the pulmonary lesions observed in the chest CT suggest reactivation of a quiescent TB. Control of TB infections depends on CD4 T cells and their cytokines, interferon gamma, interleukin 12, and tumour necrosis alpha [4–7]. Different studies reported a higher incidence of TB in haematologic malignancies than in other solid tumours [8–11]. This could be explained by deeper cellular immunity suppression in these patients, due to the underlying malignancy or the treatment. Further support for this fact comes from the lower incidence of TB in autologous HSCT compared to allogeneic HSCT recipients, who present a more lasting defect in T-cell function [1].

Receiving treatment with fludarabine or an allograft from an unrelated mismatched donor, as in our case, has been reported as a predisposing factor [12, 13]. Other risk factors, such as conditioning therapies including busulphan, cyclophosphamide or total body irradiation, receiving a T-cell depleted allograft or developing graft-versus-host disease, were not present in our patient [14, 15].

This case presents two characteristics that challenge the diagnosis. First, isolated lymph node involvement is uncommon in post-transplant TB. Russo *et al* extensively reviewed 25 studies, published from 1980 to 2009, which included patients who developed TB after HSCT [1]. The time of presentation was late, in contrast to our patient, with a median of 257 (21–1410) days from the HSCT to the diagnosis, the lung was the most commonly involved organ and they only identified a study describing a patient with lymph node TB [16]. Second, the presence of synchronous EBV replication in combination with lymph node enlargement can be present in post-transplant lymphoproliferative disorders (PTLD). The majority of PTLD in allogeneic HSCT are associated with EBV infection and develop within the first six months after transplant. Diagnosis is based on histologic findings, which range from polyclonal B-cell proliferations to overt malignant lymphomas [17]. Moreover, it has been reported the coexistence of both complications, nodal TB and EBV-PTLD, in one allogeneic HSCT recipient from Germany [18].

Whether or not screening tests should be routinely used to identify latent TB in HSCT candidates is still controversial. TST is the most widely used test to identify latent TB, but it lacks sensitivity in immunocompromised patients. Interferon gamma release assays, such as Quantiferon-TB, have recently shown efficacy in predicting the development of TB in HSCT recipients in whom latent TB was not detected by TST, emerging as a promising alternative for the future [19].

## Conclusion

This case illustrates that, even in non-endemic countries, we must keep a high suspicion index in order to make an early diagnosis and an adequate treatment of TB in HSCT patients, especially in those with risk factors.

## Conflicts of interest

The authors declare that they have no conflicts of interest.

## Authors’ contributions

Guillermo Martín-Sánchez wrote the article. Marta Drake-Pérez and José Luis Rodriguez obtained the figures and critically revised the manuscript. Lucrecia Yañez had the original idea and critically revised the manuscript. Andrés Insunza and Carlos Richard critically revised the manuscript. All authors read and approved the final manuscript.
